# Evaluating the progress to eliminate mother-to-child transmission (MTCT) of syphilis in Hunan Province, China: A study based on a health service delivery model

**DOI:** 10.1371/journal.pone.0203565

**Published:** 2018-09-07

**Authors:** Zhiyu Liu, Tingting Wang, Yuan Liu, Aihua Wang, Donghua Xie, Fanjuan Kong, Lili Xiong, Lizhang Chen, Hua Wang

**Affiliations:** 1 Information Management Division, Hunan Provincial Maternal and Child Health Care Hospital, Changsha, Hunan Province, China; 2 Department of Epidemiology and Health Statistics, Xiangya School of Public Health, Central South University, Changsha, Hunan Province, China; 3 Department of Chronic Disease Control and Prevention, Hunan Provincial Center for Disease Control and Prevention, Changsha, Hunan Province, China; 4 Division of Medical Genetics, Hunan Provincial Maternal and Child Health Care Hospital, Changsha, Hunan Province, China; University of Helsinki, FINLAND

## Abstract

To prevent mother-to-child transmission (MTCT) of syphilis, Hunan Province launched a free syphilis screening and treatment programme in 2011. Thus far, the programme has been implemented for 6 years. This study aimed to assess progress toward the elimination of MTCT of syphilis in Hunan Province from 2011–2016. Estimates of syphilis-related adverse pregnancy outcomes (APOs) were based on the health service delivery model developed by the WHO, which were then translated into disability-adjusted life years (DALYs). Default values in the model were replaced by a Chinese version. The progress of this programme was assessed through the reduction of estimated DALYs with and without screening and treatment services. The results showed that the estimated number of syphilis-related APOs in Hunan Province from 2011 to 2016 was 3,840, more than 70% of which occurred among women who had at least one antenatal care visit but were not screened or treated for syphilis during pregnancy. The public health burden resulting from maternal syphilis-related APOs was 192,528 DALYs over six years, and with the current screening and treatment coverage, approximately 163,794 expected DALYs (46%) were averted. Our estimates indicate that in Hunan Province, syphilis in pregnancy continues to be an important cause of APOs, which can lead to substantial perinatal morbidity and mortality. Approximately half of the expected public health burden resulting from syphilis-related APOs was averted by the current screening and treatment services, which suggests progress toward the elimination of MTCT of syphilis in Hunan Province.

## Introduction

Syphilis in pregnancy can induce adverse pregnancy outcomes (APOs), including stillbirth, preterm birth, low birth weight, neonatal death and congenital infection among infants [1]. Globally, nearly 1.0 million cases of syphilis occur among pregnant women in 2012, a high proportion of which are untreated or inadequately treated [2]. Nearly half of women with untreated syphilis can experience catastrophic foetal outcomes, including an estimated 205,000 perinatal deaths in 2012 globally [2]. According to the national sexually transmitted disease surveillance system, the incidence of congenital syphilis was 0.01 cases per 100,000 live births in 1991, which has increased dramatically to 69.9 cases per 100,000 live births in 2013 in China [3, 4].

APOs associated with maternal syphilis can be effectively prevented by screening pregnant women early in pregnancy and by the timely treatment of those who were diagnosed as syphilis-seropositive. A single dose of long-acting penicillin can effectively prevent syphilis-related APOs [5]. Depending on the disease stage, maternal syphilis can be effectively treated by giving one-dose (primary and secondary syphilis) or three doses (latent syphilis) of long-acting penicillin [6]. It is estimated that the cost of screening and treatment for syphilis is less than 1 US dollar per test and 1 US dollar per penicillin dose, respectively [7]. Health economists estimate that screening and treatment programme for syphilis are one of the most cost-effective public health interventions available [7], and antenatal syphilis screening and treatment is clearly cost-effective, even in the settings with prevalence rates below 1% [7–10].

In 2007, the WHO launched a global initiative to eliminate congenital syphilis [7]. Responding to the call of the WHO, in 2010, the former Chinese Ministry of Health formulated the *National Program for Prevention and Control of Syphilis Program (2010–2020)* [11], with the aim of preventing mother-to-child transmission (MTCT) of syphilis and reducing and eventually eliminating congenital syphilis through 10 years of effort. In 2011, with support of national special funding, Hunan Province launched the programme of preventing MTCT of syphilis which was a part of the strategy for triple elimination of MTCT of human immunodeficiency virus (HIV), syphilis, and viral hepatitis, and so far, the programme has been implemented for 6 years. To effectively assess progress in the elimination of MTCT of syphilis in Hunan Province and to guide policy and advocacy efforts, data on the burden of syphilis in pregnancy and associated APOs are needed. However, even in developed countries with robust laboratory infrastructure, it is difficult to definitively diagnose MTCT of syphilis [12]. Moreover, due to the missing demographic data and high crowd mobility, the number of women with syphilis in pregnancy and associated APOs in Hunan province reported by the surveillance sites may be lower than the actual number, which leads to an underestimation of the burden of syphilis in pregnancy and associated APOs. Thus, estimates of the burden of disease in the area must rely on modelled data.

In 2013, based on a health service delivery model, the WHO reported 2008 global estimates of pregnant women with probable active syphilis and associated APOs [12]. It was estimated that there were 1.36 million cases of maternal syphilis, and 0.52 million syphilis-related APOs occurred globally in 2008 [12]. This model not only took the utilization of antenatal care (ANC) service into account but also removed background mortality and morbidity (i.e., expected APOs that could occur in pregnant women without syphilis).

To assess progress in the elimination of MTCT of syphilis in Hunan Province, the province-specific data on syphilis-related APOs and the standard measures of disease burden (e.g., disability-adjusted life years (DALYs)) associated with this condition are needed. These data can also be used to guide local policy and advocacy efforts and to facilitate optimal resource allocation, which will help to strengthen maternal and child health services.

In this study, we estimated the disease burden resulting from syphilis-related APOs in Hunan Province from 2011 to 2016 and assessed the progress made in the prevention programme.

## Materials and methods

The primary objective of this analysis was to assess the progress made in the programme of preventing MTCT of syphilis in Hunan Province, which was achieved through the estimation of three values: the number of syphilis-related APOs, the estimated number of APOs in the form of DALYs, and the reduction in DALYs achieved by screening and treatment services. The secondary objective was to calculate the number of DALYs averted in the optimal situation in which the coverage of syphilis screening and treatment services has met the WHO-proposed targets for eliminating MTCT of syphilis (i.e., coverage of syphilis testing of pregnant women ≥ 95% and treatment of syphilis-seropositive pregnant women ≥ 95%).

### Part 1 Estimate the number of syphilis-related APOs in Hunan Province

#### Estimation model

In this analysis, we estimated the number of syphilis-related APOs via the health service delivery model, which was developed by the WHO and reviewed and approved by the Child Health Epidemiology Reference Group. This estimation included calculations of the number of pregnant women with syphilis infection and the number of syphilis-related APOs. Details of the model have been described elsewhere [12]. In this model, syphilis infection was defined as seropositivity on both the Treponema pallidum (TP) and non-TP tests. The number of pregnant women with syphilis infection was calculated as the product of the prevalence of syphilis in pregnant women and number of pregnant women. The number of syphilis-related APOs was calculated as the product of the number of pregnant women with syphilis infection and the probability of those women having syphilis-related APOs (the difference between the probability of these women having APOs and the probability of the general population of women having the same APOs).

#### Estimation process and data sources

**Fig 1:**
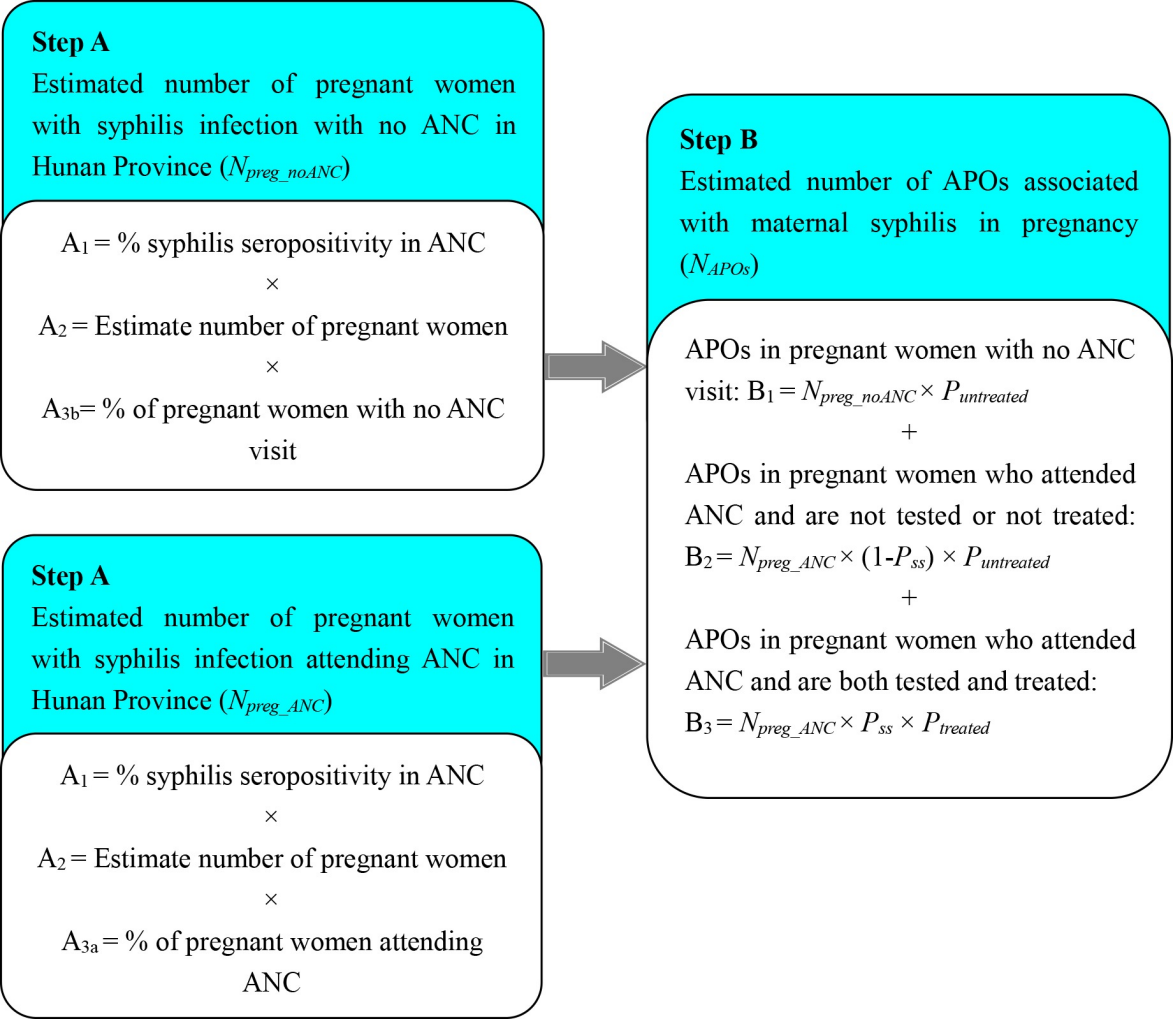
Flowchart of model to estimate the number of syphilis-related adverse pregnancy outcomes in Hunan Province. ANC, antenatal care.

Based on the health service delivery model, this estimation involved two steps (Fig 1): calculation of the number of pregnant women with syphilis infection in Hunan Province and calculation of the number of syphilis-related APOs in Hunan Province.

For Step A, we estimated the number of pregnant women in Hunan Province with syphilis infection with no antenatal care (ANC) and with at least one ANC visit.

$$N_{preg\_noANC}=P_{ss}\times N_{preg}\times P_{preg\_noANC}$$

$$N_{preg\_ANC}=P_{ss}\times N_{preg}\times P_{preg\_ANC}$$

Data on the syphilis seroprevalence among ANC attendees (*P*_*ss*_, Step A_1_ in Fig 1) in Hunan Province were obtained through China’s Information System of Prevention of Mother-to-Child Transmission of Syphilis Management, a nationwide health facility-based case report system used to monitor and evaluate the prevalence of syphilis infection in mother and their offspring in China. Maternal syphilis is monitored through mandatory case reporting by medical and health institutions in 31 provinces, municipalities, and autonomous regions in China, including general hospitals, maternal and children’s hospitals and other health agencies across the country. Diagnosis of maternal syphilis was based on the standard of health industry in the People's Republic of China (WS 273–2007) [13]. All women registered in the system were seropositive in both TP and non-TP tests, regardless of specific test methods. We assumed that the percentage of women with at least one ANC visit (*P*_*preg_ANC*_, Step A_3a_ in Fig 1) was equal to the antenatal examination rate, which was available from the *Hunan Province’s Annual Report of Maternal and Child Health*. Thus, the number of pregnant women (*N*_*preg*_, Step A_2_ in Fig 1) were estimated as the number of women attending their first ANC services divided by the antenatal examination rate.

For Step B, we estimated the number of syphilis-related APOs among syphilis-infected women who had no ANC visits.

$$N_{APOs\_noANC}=N_{preg\_noANC}\times P_{untreated}$$

We also estimated the number of syphilis-related APOs among syphilis-infected women who had at least one ANC visit and were not tested or not treated (if seropositive), and were both tested and treated.

$$N_{APOs\_ANC}=N_{preg\_ANC}\times (1-P_{\mathrm{tt}})\times P_{untreated}+N_{preg\_ANC}\times P_{\mathrm{tt}}\times P_{treated}$$

APOs included spontaneous abortion, stillbirth, preterm birth/low birth weight, neonatal death and congenital syphilis. The proportion of pregnant women who were both tested and treated for syphilis (*P*_*tt*_) was calculated as the product of the syphilis screening rate and the syphilis treatment rate which were available from the *Hunan Province’s Annual Report of Maternal and Child Health*. Since there was no nationwide or representative data available in China, in this study, we conducted a meta-analysis to estimate the summary probabilities of untreated syphilis-related APOs (*P*_*untreated*_), as well as the summary probabilities of treated syphilis-related APOs (*P*_*treated*_) occurring in Chinese pregnant women. Details of the methods and results of the meta-analysis were shown in S1 File. According to our meta-analysis (S1 File), *P*_*untreated*_ for each adverse outcome category was 12.6% for spontaneous abortion, 20.0% for stillbirth, 12.9% for preterm birth/low birth weight, 8.0% for neonatal death, and 34.0% for congenital syphilis; *P*_*treated*_ for each adverse outcome category was 3.6% for spontaneous abortion, 1.7% for stillbirth, 2.8% for preterm birth/low birth weight, 0.2% for neonatal death, and 8.8% for congenital syphilis.

Year-specific data inputs are displayed in Table 1.

**Table 1:** Year-specific data inputs.

Year	Antenatal Examination Rate (%)	At least one ANC Visit	ANC Syphilis Screening Rate (%)	ANC Syphilis Treatment Rate (%)	Syphilis seroprevalence (‰)
2011	94.58	826,731	58.34	42.41	1.41
2012	95.24	900,105	70.88	63.62	1.25
2013	96.00	874,732	80.60	71.82	1.24
2014	95.90	918,863	85.41	75.41	1.43
2015	96.12	917,076	87.21	77.88	1.28
2016	96.82	984,444	90.11	82.96	1.71

ANC, antenatal care.

### Part 2 Translated the estimated number of APOs into DALYs

The DALYs are a comprehensive index that measures health loss due to both fatal and non-fatal disease burden. One DALY can be considered as one year of ‘healthy’ life lost [14]. In this analysis, we translated the estimated number of APOs into DALYs according to the methodology outline in the report of the WHO. For a specific cause (i.e., a disease or health condition), DALYs are calculated as the sum of the years of life lost (YLLs) due to premature mortality caused by that cause and the years of life lived with disability (YLDs) for people living in states of less than optimal health resulting from the specific cause [14]. The YLLs due to spontaneous abortion, stillbirth and neonatal death were calculated as the product of the neonatal standard loss function of 86.01 years and the number of incremental cases, calculated by the health service delivery model. To estimate the YLDs, the number of incremental cases were multiplied by the average duration of the disease (i.e., local life expectancy at birth) and its disability weights, which reflect the severity of the disease on a scale from 0 (perfect health) to 1 (dead). Based on the Global Burden of Disease 2004 Update, the disability weights were 0.106 for low birth weight (all sequelae) and 0.315 for congenital syphilis [15]. Since there was no evidence of disability weight for preterm birth, we set the disability weight of preterm birth/low birth weight equal to 0.106 as an alternative.

### Part 3 Estimate the reduction in DALYs achieved by screening and treatment service

In this analysis, three screening and treatment coverage scenarios, including scenarios without any screening and treatment services for syphilis, scenarios of current screening and treatment coverage, and scenarios with coverage to meet the WHO-proposed targets for eliminating MTCT of syphilis, were used as the baseline, current, and best-case scenarios, respectively, to calculate the estimated DALYs resulting from adverse outcomes associated with maternal syphilis. The estimated reduction in DALYs achieved by screening and treatment services was calculated by subtracting the estimated DALYs in the current or best-case scenarios from the estimated DALYs in the baseline scenarios.

## Results

Year-specific model results are displayed in Table 2. From 2011 to 2016, the estimated number of APOs associated with maternal syphilis in Hunan Province were 876, 676, 548, 600, 506 and 634, respectively, with a total of 3,840 cases over the six years. Approximately 74% (2,817) of these cases occurred among women who had at least one ANC visit but were not screened or treated for syphilis during pregnancy. The estimated numbers for each adverse outcome category over the six years were as follows: 602 cases of spontaneous abortion, 784 cases of stillbirth, 577 cases of preterm birth/low birth weight, 292 cases of neonatal death, and 1,585 cases of congenital syphilis.

**Table 2:** Estimated number of adverse pregnancy outcomes associated with maternal syphilis in Hunan Province from 2011 to 2016.

Year	Subgroup	Spontaneous abortion (n)	Stillbirth (n)	Preterm birth/low birth weight (n)	Neonatal death (n)	Congenital syphilis (n)	All adverse pregnancy outcomes (n)
**2011**		**129**	**194**	**130**	**76**	**347**	**876**
	Pregnant women with no ANC visit	8	13	9	5	23	58
	Pregnant women with both screening and treatment for syphilis	10	5	8	1	25	49
	Pregnant women without screening or treatment for syphilis	111	176	113	70	299	769
**2012**		**103**	**146**	**100**	**54**	**273**	**676**
	Pregnant women with no ANC visit	7	11	7	4	19	48
	Pregnant women with both screening and treatment for syphilis	18	9	14	1	45	87
	Pregnant women without screening or treatment for syphilis	78	126	79	49	209	541
**2013**		**87**	**111**	**83**	**42**	**225**	**548**
	Pregnant women with no ANC visit	6	9	6	4	15	40
	Pregnant women with both screening and treatment for syphilis	23	11	18	1	55	108
	Pregnant women without screening or treatment for syphilis	58	91	59	37	155	400
**2014**		**96**	**118**	**91**	**43**	**252**	**600**
	Pregnant women with no ANC visit	7	11	7	4	19	48
	Pregnant women with both screening and treatment for syphilis	30	14	24	2	74	144
	Pregnant women without screening or treatment for syphilis	59	93	60	37	159	408
**2015**		**82**	**98**	**76**	**36**	**214**	**506**
	Pregnant women with no ANC visit	6	9	6	4	16	41
	Pregnant women with both screening and treatment for syphilis	29	14	22	2	70	137
	Pregnant women without screening or treatment for syphilis	47	75	48	30	128	328
**2016**		**105**	**117**	**97**	**41**	**274**	**634**
	Pregnant women with no ANC visit	7	11	7	4	19	48
	Pregnant women with both screening and treatment for syphilis	45	21	35	3	111	215
	Pregnant women without screening or treatment for syphilis	53	85	55	34	144	371
**Total**		**602**	**784**	**577**	**292**	**1,585**	**3,840**
	Pregnant women with no ANC visit	41	64	42	25	111	283
	Pregnant women with both screening and treatment for syphilis	155	74	121	10	380	740
	Pregnant women without screening or treatment for syphilis	406	646	414	257	1,094	2,817

ANC, antenatal care.

Table 3 showed the public health burden resulting from APOs associated with maternal syphilis in Hunan Province from 2011 to 2016. The total estimated number of syphilis-related APOs were translated into 192,528 DALYs, with an annual average of 32,088 DALYs. The specific breakdown of DALYs over the six years by outcome (% of total) was 51,778 DALYs (26.9%) for spontaneous abortion, 67,432 DALYs (35.0%) for stillbirth, 5,260 DALYs (2.7%) for preterm birth/low birth weight, 25,115 DALYs (13.0%) for neonatal death, and 42,943 DALYs (22.3%) for congenital syphilis. Approximately 76% (146,002 DALYs) of the total DALYs over the six years occurred among women who had at least one ANC visit but were not screened or treated for syphilis during pregnancy.

**Table 3:** Estimated public health burden in terms of DALYs resulting from adverse pregnancy outcomes associated with maternal syphilis in Hunan Province from 2011 to 2016. (DALYs).

Year	Subgroup	Spontaneous Abortion	Stillbirth	Preterm birth/low birth weight	Neonatal death	Congenital syphilis	All Adverse pregnancy Outcomes
**2011**		**11,095**	**16,686**	**1,185**	**6,537**	**9,401**	**44,905**
	Pregnant women with no ANC visit	688	1,118	82	430	623	2,942
	Pregnant women with both screening and treatment for syphilis	860	430	73	86	677	2,126
	Pregnant women without screening or treatment for syphilis	9,547	15,138	1,030	6,021	8,101	39,837
**2012**		**8,859**	**12,558**	**912**	**4,645**	**7,397**	**34,369**
	Pregnant women with no ANC visit	602	946	64	344	515	2,471
	Pregnant women with both screening and treatment for syphilis	1,548	774	128	86	1,219	3,755
	Pregnant women without screening or treatment for syphilis	6,709	10,837	720	4,215	5,663	28,143
**2013**		**7,483**	**9,547**	**757**	**3,612**	**6,096**	**27,495**
	Pregnant women with no ANC visit	516	774	55	344	406	2,095
	Pregnant women with both screening and treatment for syphilis	1,978	946	164	86	1,490	4,665
	Pregnant women without screening or treatment for syphilis	4,989	7,827	538	3,182	4,199	20,735
**2014**		**8,257**	**10,149**	**830**	**3,698**	**6,828**	**29,762**
	Pregnant women with no ANC visit	602	946	64	344	515	2,471
	Pregnant women with both screening and treatment for syphilis	2,580	1,204	219	172	2,005	6,180
	Pregnant women without screening or treatment for syphilis	5,075	7,999	547	3,182	4,308	21,111
**2015**		**7,053**	**8,429**	**693**	**3,096**	**5,798**	**25,069**
	Pregnant women with no ANC visit	516	774	55	344	434	2,122
	Pregnant women with both screening and treatment for syphilis	2,494	1,204	201	172	1,897	5,968
	Pregnant women without screening or treatment for syphilis	4,043	6,451	438	2,580	3,468	16,979
**2016**		**9,031**	**10,063**	**884**	**3,526**	**7,424**	**30,928**
	Pregnant women with no ANC visit	602	946	64	344	515	2,471
	Pregnant women with both screening and treatment for syphilis	3,871	1,806	319	258	3,007	9,261
	Pregnant women without screening or treatment for syphilis	4,559	7,311	501	2,924	3,901	19,197
**Total**		**51,778**	**67,432**	**5,260**	**25,115**	**42,943**	**192,528**
	Pregnant women with no ANC visit	3,527	5,505	383	2,150	3,007	14,572
	Pregnant women with both screening and treatment for syphilis	13,332	6,365	1,103	860	10,295	31,955
	Pregnant women without screening or treatment for syphilis	34,920	55,563	3,774	22,105	29,640	146,002

ANC, antenatal care; DALYs, disability adjusted life years.

**Fig 2:**
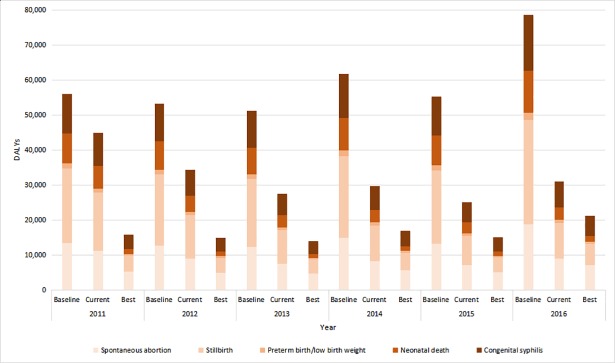
Estimated number of syphilis-related adverse pregnancy outcomes in the baseline, current, and best-case scenarios of testing and treatment in Hunan Province from 2011 to 2016.

Fig 2 shows the estimated DALYs resulting from APOs associated with maternal syphilis between 2011 and 2016. In the baseline scenario without any screening or treatment services, there would be 356,320 DALYs resulting from APOs associated with maternal syphilis over the six years. From 2011 to 2016, using the current scenario for syphilis screening and treatment, approximately 163,794 DALYs (46%) were averted, including 33,458 DALYs for spontaneous abortion, 67,861 DALYs for stillbirth, 3,984 DALYs for preterm birth/low birth weight, 28,986 DALYs for neonatal death, and 29,505 DALYs for congenital syphilis (Table 4). By year, the DALYs averted in the current scenario were estimated as 11,102 DALYs in 2011, 18,966 DALYs in 2012, 23,632 DALYs in 2013, 32,092 DALYs in 2014, 30,267 DALYs in 2015, and 47,735 DALYs in 2016. If the WHO-proposed targets for eliminating MTCT of syphilis, that is, if at least 95% screening and 95% treatment had been reached between 2011 and 2016, then 258,355 DALYs (73%) could have been averted potentially: 40,142 DALYs in 2011, 38,465 DALYs in 2012, 37,095 DALYs in 2013, 44,889 DALYs in 2014, 40,281 DALYs in 2015, and 57,843 DALYs in 2016 (Table 4).

**Table 4:** Estimated DALYs due to adverse outcomes associated with maternal syphilis averted in the current scenario, as well as in the best case scenario between 2011 to 2016 (DALYs).

	DALYs averted in the current scenario						DALYs averted in the best case scenario					
	Spontaneous abortion	Stillbirth	Preterm birth/low birth weight	Neonatal death	Congenital syphilis	All adverse pregnant outcomes	Spontaneous abortion	Stillbirth	Preterm birth/low birth weight	Neonatal death	Congenital syphilis	All adverse pregnant outcomes
2011	2,323	4,558	265	1,978	1,978	11,102	8,257	16,599	967	7,139	7,180	40,142
2012	3,870	7,741	474	3,440	3,441	18,966	7,826	15,997	939	6,795	6,908	38,465
2013	4,730	9,891	574	4,129	4,308	23,632	7,568	15,396	902	6,537	6,692	37,095
2014	6,537	13,332	775	5,677	5,771	32,092	9,117	18,578	1,094	7,999	8,101	44,889
2015	6,193	12,557	738	5,333	5,446	30,267	8,257	16,685	966	7,139	7,234	40,281
2016	9,805	19,782	1,158	8,429	8,561	47,735	11,697	23,824	1,404	10,235	10,323	57,483
Total	33,458	67,861	3,984	28,986	29,505	163,794	52,722	107,079	6,272	45,844	46,438	258,355

DALYs, disability adjusted life years.

## Discussion

As we know, due to the paucity of accurate and reliable demographic and epidemiological data, it is hard to accurately calculate the public health burden due to APOs associated with maternal syphilis in China, even at the subnational level. For the first time, this analysis produced subnational estimations of the public health burden associated with syphilis-related APOs in China based on a mathematical model and determined the extent of progress made in the programme of preventing MTCT of syphilis in Hunan Province through comparison analysis.

In this analysis, we adopted the health service delivery model to estimate the number of pregnant women with syphilis infection, as well as the number of associated APOs in Hunan Province. This model took the existing screening and treatment service into account and removed background morbidity and mortality. The number of APOs calculated by the model were attributable to maternal syphilis infection, which truly reflected the effect of syphilis infection on the foetus and neonates. Furthermore, the model allowed us to replace the default values with more appropriate data, if it were available. Since there is no evidence of whether or not maternal syphilis has the same effect on pregnancy outcomes among Chinese and non-Chinese women, we integrated and analysed the related data to establish the extent of syphilis-related APOs among women with and without penicillin treatment in China through meta-analysis (S1 File). The results suggested that the summary estimates of the proportion of APOs due to untreated maternal syphilis among Chinese women (20.0% for stillbirth, 8.0% for neonatal death, 12.9% for preterm birth or low birth weight, and 34.0% for congenital syphilis) were inconsistent with those among non-Chinese women (21.0% for stillbirth or early foetal death, 9.3% for neonatal death, 5.8% for preterm birth or low birth weight, and 15.5% for congenital syphilis [16]). In addition, the default values of the effectiveness (*E*) of syphilis screening and penicillin treatment in averting APOs in the model were derived from the meta-analysis conducted in pregnant women with active syphilis (i.e., rapid plasma reagin titre ≥ 1:8) [5]. However, the model had not taken the baseline non-TP titre of pregnant women into account when calculating, which might lead to underestimation of the number of syphilis-related APOs. Moreover, in that meta-analysis, all pregnant women in the intervention arm received at least 2.4 million units of penicillin at least 28 days prior to delivery [5]. Nevertheless, the model did not involve the treatment time when estimating the number of pregnant women who were both tested and treated for syphilis during pregnancy, given that current studies demonstrated a time length between the completion of the first penicillin treatment and delivery of less than 28 days as a risk factor for syphilis-related APOs [17, 18], which might lead to an underestimate in the number of syphilis-related APOs as well. Therefore, in this analysis, we used the data derived from our meta-analysis (S1 File) to calculate the number of syphilis-related APOs in Hunan Province instead of the default values given in the model.

In addition, as recommended, the number of pregnant women was calculated as the sum of the number of live births, stillbirths (after 28 week gestation) and early foetal deaths (22–28 weeks gestation), which did not include first trimester losses (miscarriages) [12]. However, due to the absence of the estimates of early foetal deaths in China and the fact that among women who have had at least one visit to ANC in China, 70% had their first ANC during early pregnancy (≤ 12 gestational weeks) [19], we could not estimate the number of pregnant women in Hunan Province by the recommended method. As an alternative, we assumed that the percentage of women with at least one ANC visit was equal to the antenatal examination rate used to calculate the number of pregnant women in Hunan Province which theoretically covered individuals who had miscarriages. Therefore, when calculating the number of syphilis-related APOs, we included the category of spontaneous abortions.

Based on the health service delivery model, our estimates suggested that over 3,800 syphilis-related adverse pregnant outcomes occurred between 2011 and 2016, of which approximately 600 were spontaneous abortion, 780 were stillbirth, 580 were preterm birth/low birth weight, 290 were neonatal death and 1,580 were congenital syphilis. These results indicate that syphilis continues to be an important cause of substantial perinatal morbidity and mortality. Additionally, it is worth noting that this analysis does not include the additional infant deaths that may occur after the first month of life due to low birth weight, preterm birth, and syphilis infection in new-borns. As estimated by McDermott et al. [20], these events may result in approximately 10% additional infant deaths by 1 year. These findings indicate that preventing MTCT of syphilis is not only an effective way to reduce perinatal morbidity and mortality but also an important measure to decrease infant mortality during the first year of life. In addition, in this analysis, approximately 74% of the syphilis-related APOs occurred among women who had at least one ANC visit but were not tested or treated for syphilis if seropositive. This findings highlights the need to improve the quality of ANC while ensuring universal access to early ANC.

In this analysis, we found that approximately 192,528 DALYs occurred in Hunan Province between 2011 and 2016, with an average of 32,088 DALYs per year, which equated to 72% of DALYs associated with tuberculosis in the province in 2005 [21], 36% of DALYs associated with cardiovascular disease in the province in 2011 [22], and 30% of DALYs associated with HIV infection and acquired immunodeficiency syndrome (AIDS) throughout the country in 2007 [23]. These figures suggested that syphilis-related APOs had brought a heavy public health burden upon the society that even exceeded the burden of HIV/AIDS; this finding reminded policymakers charged with resource allocation to regard the elimination of MTCT of syphilis as a public health priority.

Existing screening and treatment services were able to avert nearly half of the expected public health burden resulting from syphilis-related adverse pregnancy outcomes in Hunan Province between 2011 and 2016; if WHO-proposed targets for eliminating MTCT of syphilis for screening and treatment had been met over the six years, more than 70% of the expected burden could have been averted. However, even though the target had been reached, a certain number of syphilis-related APOs still occurred each year, and the incidence of congenital syphilis was significantly higher than 50/100,000 live births. Therefore, further studies are needed to determine how many cases of congenital syphilis and what level of health service delivery are needed to achieve the ultimate goal of eliminating congenital syphilis as a public health problem. In addition, given that maternal syphilis infection is the primary cause of syphilis-related APOs, and that screening and treatment services for preventing MTCT of syphilis is not 100% effective, the prevention of maternal syphilis infection should also be taken as an important strategy to achieve the goal of full elimination of congenital syphilis. Due to the dynamics of syphilis transmission, the prevalence of syphilis among both the general and high-risk populations affects the prevalence of syphilis among pregnant women [24]. Therefore, to prevent pregnant women from syphilis infection, as well as to avert syphilis-related APOs fundamentally, more health resources should be invested into the prevention and control of syphilis in Hunan Province. Meanwhile, target interventions for the general and high-risk populations are necessary to improve control of the elimination of MTCT of syphilis. In addition, although the National Health and Family Planning Commission of the People’s Republic of China demands medical and health institutions to provide services such as notification and testing for partners of pregnant women with confirmed syphilis infection, more than 50% of these partners were not tested for syphilis in Hunan Province. This poor enforcement may be an obstacle to the elimination of MTCT of syphilis. ANC workers should improve the testing for syphilis among partners of infected women and ensure adequate and prompt treatment for both infected women and their partners.

Although the proportions of syphilis screening and treatment during pregnancy have increased each year in Hunan Province since the implementation of the programme to prevent MTCT of syphilis, the DALYs caused by maternal syphilis infection increased in 2016 due to the increase in the number of pregnant women with syphilis infection. This trend further emphasizes the importance of preventing pregnant women from acquiring syphilis infection.

Our estimates are subject to some limitations. On the one hand, since there was neither a national or regional record of miscarriages nor a percentage of pregnant women with at least one ANC visit, we presumed that the percentage of pregnant women with at least one ANC visit was equal to the antenatal examination rate as an alternative to estimate the number of pregnant women, which might lead to a miscalculation. However, due to the limitation of information, we could not estimate the error caused by the assumption. On the other hand, in this analysis, when calculating the number of syphilis-related APOs based on the health service delivery model, we used the data derived from our meta-analysis (S1 File), which was considered to be more appropriate than the default values given in the model. Theoretically, we should compare the estimated number with case reports to see if there are significant differences and to verify whether the substitution is appropriate. However, due to the lack of appropriate valid data, we could not complete this task. Finally, since there was no data on the disability weight for preterm birth at present, we took that of low birth weight as an alternative, the calculation of which might be inconsistent with the actual disability weight. These limitations highlight the need to improve the quality of the data through strengthening the national monitoring and surveillance systems.

## Conclusions

Our estimates indicate that syphilis in pregnancy continues to be an important cause of APOs, which can lead to substantial perinatal morbidity and mortality in Hunan Province. Approximately half of the expected public health burden resulting from syphilis-related APOs was averted by the current screening and treatment services, which suggests progress towards the elimination of MTCT of syphilis in Hunan Province. Nonetheless, untreated maternal syphilis remains a substantial cause of preventable perinatal morbidity and mortality, even in women receiving ANC. Improving access to quality antenatal care, including syphilis testing and treatment, and ensuring adequate and prompt treatment for infected women and their partners are all important for achieving the elimination of MTCT of syphilis.

## Supporting information

S1 File Meta-analyses of the proportions of adverse pregnancy outcomes among women with treated and untreated syphilis. Contains the PRISMA Checklist.
(DOC)
