# Supplementary material for: Evaluating the progress to eliminate mother-to-child transmission (MTCT) of syphilis in Hunan Province, China: A study based on a health service delivery model
Source: PLoS One. 2018 Sep 7;13(9):e0203565. doi: 10.1371/journal.pone.0203565 (PMC6128588; doi:10.1371/journal.pone.0203565)
Supplement: S1 File — Contains the PRISMA Checklist. (DOC) [file pone.0203565.s001.doc]

**S1 File. Meta-analyses of the proportions of adverse pregnancy outcomes among women with treated and untreated syphilis**

**1 Methods**

**1.1 Data sources and search strategy**

We searched the PubMed, Web of Science, EMBASE, China National Knowledge Infrastructure (CNKI), Wanfang, Weipu and China Biology Medicine (CBM) databases from inception to January 1, 2017, for articles assessing adverse pregnancy outcomes (APOs) in women with syphilis in China. The search terms used were as follows: syphilis, maternal, pregnancy, fetal, congenital, mother-to-child transmission, MTCT, pregnancy outcome, fetal death, stillbirth, preterm, low birth weight, neonatal death, perinatal death, perinatal mortality. Search strategy details in PubMed are shown in Table 1. The language of the searched literature was restricted to Chinese and English. In addition, a manual search was performed of the reference lists of all articles selected in the first step. The entire process was independently completed by two researchers.

**1.2 Inclusion and exclusion criteria**

The objective of this study was to estimate the pooled incidence of APOs due to treated and untreated maternal syphilis, which were the difference between the pooled incidence of APOs in women with treated or untreated syphilis and these in women without syphilis. Hence, for studies that not only reported the incidence of APOs in women with syphilis, but also reported the incidence in women without syphilis, we would calculate the incidence of APOs in women with and without syphilis at the same time. Among all the APOs, we were interested in spontaneous abortion, stillbirth, preterm birth/low birth weight, neonatal death and congenital syphilis.

Studies were considered eligible for inclusion in this meta-analysis when they met the following criteria: 1) original investigations described APOs in pregnant Chinese women with syphilis infection (i.e., women who were seropositive in both Treponema pallidum and non-Treponema pallidum tests, regardless of specific test methods); 2) studies provided information about sample size and incidence estimations of target APOs in women with syphilis (or data from which these could be calculated); 3) cohort studies, experimental studies and case series; 4) articles published in English or Chinese. Conversely, studies were excluded if they 1) were conference presentations and abstracts, editorials, quantitative studies, case-control studies, review papers or duplicate publications; 2) did not specify the diagnostic criteria for maternal syphilis; 3) provided information in pregnant women with syphilis and HIV co-infection; 4) reported data from China’s Information System of Prevention of Mother-to-Child Transmission of Syphilis Management; 5) sample size < 60; 6) provided incomplete or unclear data or logical errors. When more than one study involved the same population, only the most comprehensive or more recently published one was included. In addition, if the same data were published in both English and Chinese, the paper published in Chinese was excluded.

**1.3 Data extraction**

Two reviewers independently extracted and evaluated the data from each included article using a defined data abstraction form. Disagreements were resolved via discussion or consultation with a third reviewer when consensus could not be achieved. Data was as follows: first author and year of publication, geographic location, study design, syphilis screening and diagnosis test, prevalence of syphilis in pregnancy (‰), sample size of pregnant women with treated/untreated syphilis and women without syphilis, and reported APOs.

For syphilis-seropositive pregnant women, at least one dose of intramuscular benzathine penicillin or other effective regimen is necessary for the treatment of primary or secondary syphilis in the mother and for the prevention of syphilis mother-to-child transmission (MTCT). Therefore, in this study, syphilis-infected women with at least 2.4 million units penicillin (at least a single dose of benzathine penicillin or the equivalent multiple dose schedule of shorter acting penicillin) during pregnancy were regarded as pregnant women with treated syphilis, and syphilis-infected women who had no penicillin treatment or had less than 2.4 million units penicillin were regarded as pregnant women with untreated syphilis.

**1.4 Methodological quality assessment**

For included studies with cohort or case-control designs, methodological quality was assessed using the Newcastle-Ottawa scale based on the following modules: 1) the selection of exposed and non-exposed cohorts, or of a case and a control; 2) the comparability of cohorts, or of the case and control; and 3) the ascertainment of outcome or exposure. In addition, the quality assessment scale for case series developed by the National Institute for Clinical Excellence was used.

**1.5 Statistical analysis**

All analyses were performed using R version 3.4.1 (R Foundation for Statistical Computing). In the presence of between-study heterogeneity, the pooled incidence estimates and corresponding 95% confidence intervals (CIs) were calculated using random-effects meta-analyses. Since the incidence of 0 or 1 is present in this study, the original rates were transformed using Freeman-Tukey double arcsine transformations which helped to stabilize the variances. Heterogeneity between studies was evaluated using the Cochran’s chi-squared test and I^2^ statistic. Cochran’s chi-squared test was used to assess whether the variation across studies was compatible with chance alone, and a p-value < 0.1 was considered to represent statistically significant heterogeneity. The I^2^ statistic, a quantitative indicator, was used to estimate the proportion of variance between studies due to statistical heterogeneity rather than chance (I^2^ ≤ 25% represents low heterogeneity, 26-50% represents moderate heterogeneity, 51-75% represents substantial heterogeneity, and 76-100% represents high heterogeneity). Publication bias was assessed based on Egger’s linear regression test (p < 0.05 indicates a significant difference). The chi-square test was used to examine the difference between subgroups (p < 0.05 indicates a significant difference). Preferred Reporting Items for Systematic Reviews and Meta-analysis guidelines were strictly adhered to wherever appropriate (Table 2).

**2 Results**

**2.1 Study Selection**

In total, 18,216 studies were identified after an initial search (Fig 1). After removing duplicates and screening titles and abstracts, 305 articles were potentially eligible and the full text were reviewed. After reading these articles carefully, 222 studies were excluded (eighty-four had fewer than 60 pregnant women, forty-seven lacked distinct diagnostic criteria for syphilis, thirty-nine reported data from monitoring system, sixteen did not report target outcomes, fifteen contained non-extractable data, six were case-control studies, five were review papers or conference abstracts, five represented duplicate publications, three reported data on pregnant women with HIV-syphilis coinfection, and two full text could not be obtained). Finally, a total of 83 studies [1-83] were included in the meta-analysis.


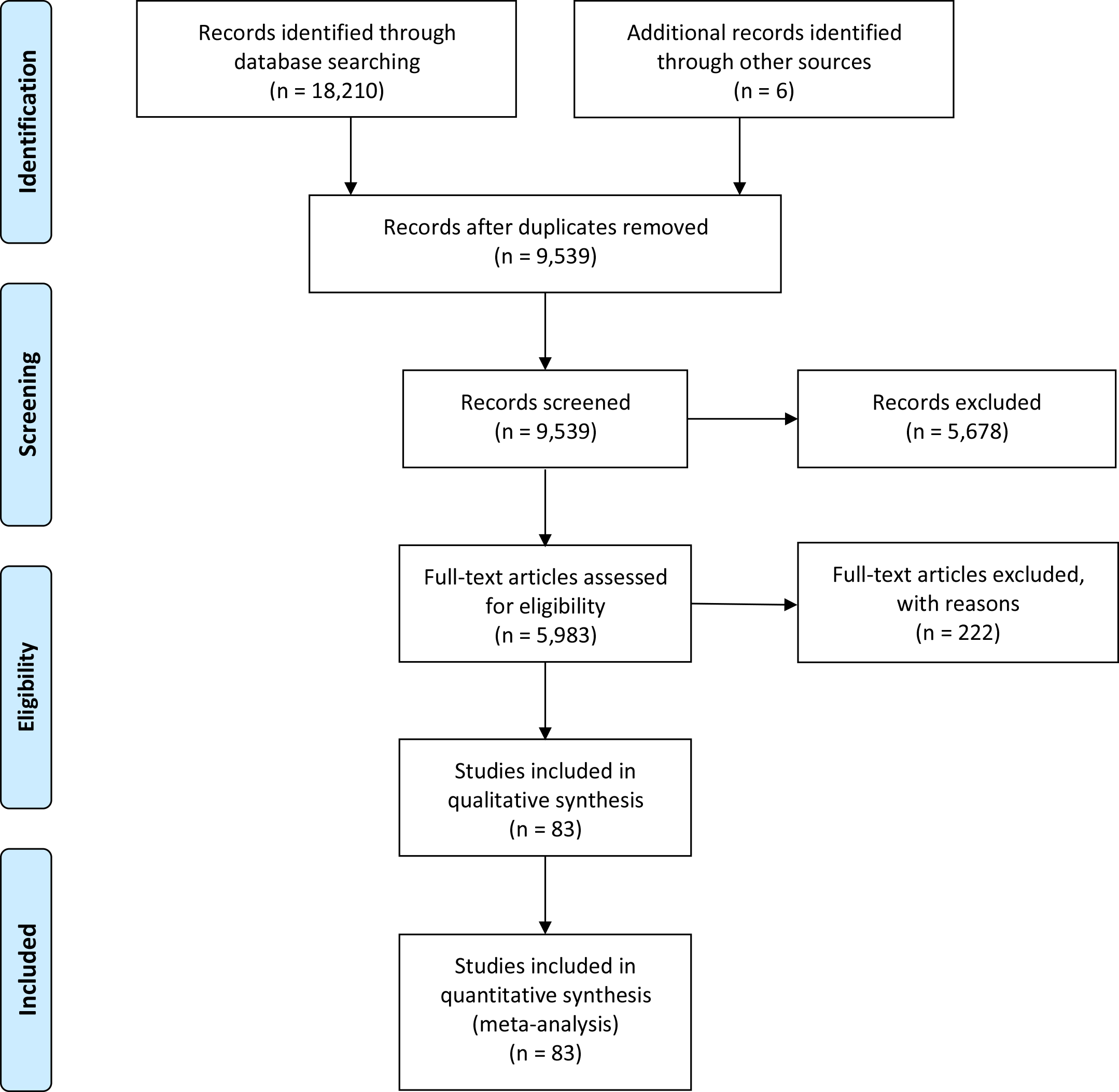


Fig 1. Flow diagram of included/excluded studies.

**2.2 Characteristics of the studies**

In the 83 studies, there were a total of 110,067 pregnant women, including 14,453 women with syphilis and 95,614 women without syphilis. The median number of participants per study was 129 (range: 60 to 50,766). Thirty-seven studies were conducted in East China, thirty-two in South China, seven in Central China, four in North China, two in Southwest and one in Northeast. The papers were published between 2001 and 2016, for which more than half were published between 2011 and 2016. All papers were observation studies, including sixty-one case series, twenty-one cohort studies (eight prospective and thirteen retrospective studies) and one case-control study. More than ninety-six percent of the studies (80/83 studies) used rapid plasma reagin and toluidine red unheated serum test as screening tests for syphilis, two studies used unheated serum reagin test and rapid plasma reagin as screening tests, and one study used only unheated serum reagin test as screening test. Expect for one study with enzyme linked immunosorbent assay as the confirmation test, the rest of the studies were confirmed by reponema pallidum particle agglutination or treponemal hemagglutination test. Twenty-eight studies reported the prevalence of syphilis among pregnant women in China, ranging from 2.74 to 64.7 cases per 1,000 pregnant women. Among the reports on the incidence among pregnant women with syphilis, eighteen (22%) studies reported the incidence of APOs among women without syphilis.

With regard to the methodological quality, none of the eighty-three studies was considered poor (< 6 points on the Newcastle-Ottawa scale or < 5 points on the quality assessment scale for case series developed by the National Institute for Clinical Excellence). Among the sixty-one case series, two scored 8 points, eighteen scored 7 points, thirty-six scored 6 points and five scored 5 points. The only case-control study scored 7 points. One of the twenty-one cohort studies scored 8 points, four scored 7 points and sixteen scored 8 points.

Among sixty-six studies reported on APOs among women with treated syphilis, twenty-two reported on the incidence of spontaneous abortion, forty-five reported on the incidence of stillbirth, forty-four reported on the incidence of preterm birth/low birth weight, nineteen reported on the incidence of neonatal death, and fifty-three reported on the incidence of congenital syphilis.

Among sixty-six studies reported on APOs among women with untreated syphilis, twenty-three reported on the incidence of spontaneous abortion, forty reported on the incidence of stillbirth, thirty-four reported on the incidence of preterm birth/low birth weight, nineteen reported on the incidence of neonatal death, and thirty-nine reported on the incidence of congenital syphilis.

**2.3 Selected APOs among women with untreated syphilis in China**

All data mentioned in this section are shown in Table 3. Among Chinese women with untreated syphilis, the pooled estimates were 13.2% (95% CI: 10.2-16.4%) for spontaneous abortion, 21.4% (95% CI: 17.7-25.3%) for stillbirth, 17.6% (95% CI: 14.7-20.8%) for preterm birth/low birth weight, 8.2% (95% CI: 4.4-12.8%) for neonatal death, and 34.0% (95% CI: 31.0-36.0%) for congenital syphilis. Significant heterogeneity was detected across studies regarding the estimates of selected APOs among women with untreated syphilis (I^2^ range: 47.5-85.8%; all p < 0.1). According to the results of Egger’s test, no evidence of publication bias was found in pooled estimates of selected APOs among women with untreated syphilis in China (all p > 0.05).

**2.4 Selected APOs among women with treated syphilis in China**

All data mentioned in this section are shown in Table 4. Among Chinese women with treated syphilis, the pooled estimates were 4.2% (95% CI: 2.9-5.8%) for spontaneous abortion, 3.1% (95% CI: 2.1-4.2%) for stillbirth, 7.5% (95% CI: 5.7-9.5%) for preterm birth/low birth weight, 0.4% (95% CI: 0.1-0.7%) for neonatal death, and 8.8% (95% CI: 6.7-11.2%) for congenital death. Significant heterogeneity was detected across studies regarding the estimates of selected APOs among women with treated syphilis (I^2^ range: 37.5-88.8%; all p < 0.1). According to the results of Egger’s test, no evidence of publication bias was found in pooled estimates of selected APOs among women with treated syphilis in China (all p > 0.05).

**2.5 Selected APOs among women without syphilis in China**

All data mentioned in this section are shown in Table 5. Among Chinese women without syphilis, the pooled estimates were 0.6% (95% CI: 0-1.6%) for spontaneous abortion, 1.4% (95% CI: 0.7-2.2%) for stillbirth, 4.7% (95% CI: 3.1-6.6%) for preterm birth/low birth weight, and 0.2% (95% CI: 3.1-6.6%) for neonatal death. Except for summary estimates of spontaneous abortion (I^2^ = 0, p = 0.446) and neonatal death (I^2^ = 0, p = 0.812), significant heterogeneity was detected across studies regarding the summary estimates of stillbirth (I^2^ = 92.4, p < 0.001) and preterm birth/low birth weight (I^2^ = 96.9, p < 0.001). According to the results of Egger’s test, no evidence of publication bias was found in pooled estimates of selected APOs among women without syphilis in China (all p > 0.05).

By calculating the differences between the pooled estimates of APOs among women with untreated syphilis and these among women without syphilis, the pooled incidence estimates of APOs due to untreated maternal syphilis were presented as follows: spontaneous abortion 12.6%, stillbirth 20.0%, preterm birth/low birth weight 12.9%, neonatal death 8.0% and congenital syphilis 34.0%. In the same way, the pooled incidence estimates of APOs due to treated syphilis were presented as follows: spontaneous abortion 3.6%, stillbirth 1.7%, preterm birth/low birth weight 2.8%, neonatal death 0.2% and congenital syphilis 8.8%.

Table 1 Search strategy used in PubMed

| Source | Search strategy |
| --- | --- |
| PubMed | 1-syphilis [Title/Abstract] |
|  | 2-pregnancy outcome [Title/Abstract] |
|  | 3-stillbirth [Title/Abstract] |
|  | 4-fetal death [Title/Abstract] |
|  | 5-neonatal death [Title/Abstract] |
|  | 6-preterm birth [Title/Abstract] |
|  | 7-low birth weight [Title/Abstract] |
|  | 8-perinatal death [Title/Abstract] |
|  | 9-perinatal mortality [Title/Abstract] |
|  | 10-maternal [Title/Abstract] |
|  | 11-pregnancy [Title/Abstract] |
|  | 12-fetal [Title/Abstract] |
|  | 13-congenital [Title/Abstract] |
|  | 14-mother to child transmission [Title/Abstract] |
|  | 15-MTCT [Title/Abstract] |
|  | 16-#1 OR #2 OR #3 OR #4 OR #5 OR #6 OR #7 OR #8 OR #9 OR #10 OR #11 OR #12 OR #13 OR #14 OR #15 |
|  | 17-#1 AND #16 |

Table 2 The PRISMA statement

| **Section/topic** | **#** | **Checklist item** | **Reported on page #** |
| --- | --- | --- | --- |
| **TITLE** | | |  |
| Title | 1 | Identify the report as a systematic review, meta-analysis, or both. | **1** |
| **ABSTRACT** | | |  |
| Structured summary | 2 | Provide a structured summary including, as applicable: background; objectives; data sources; study eligibility criteria, participants, and interventions; study appraisal and synthesis methods; results; limitations; conclusions and implications of key findings; systematic review registration number. | **NA** |
| **INTRODUCTION** | | |  |
| Rationale | 3 | Describe the rationale for the review in the context of what is already known. | **NA** |
| Objectives | 4 | Provide an explicit statement of questions being addressed with reference to participants, interventions, comparisons, outcomes, and study design (PICOS). | **NA** |
| **METHODS** | | |  |
| Protocol and registration | 5 | Indicate if a review protocol exists, if and where it can be accessed (e.g., Web address), and, if available, provide registration information including registration number. | **1** |
| Eligibility criteria | 6 | Specify study characteristics (e.g., PICOS, length of follow-up) and report characteristics (e.g., years considered, language, publication status) used as criteria for eligibility, giving rationale. | **1-2** |
| Information sources | 7 | Describe all information sources (e.g., databases with dates of coverage, contact with study authors to identify additional studies) in the search and date last searched. | **1** |
| Search | 8 | Present full electronic search strategy for at least one database, including any limits used, such that it could be repeated. | **1** |
| Study selection | 9 | State the process for selecting studies (i.e., screening, eligibility, included in systematic review, and, if applicable, included in the meta-analysis). | **1-2** |
| Data collection process | 10 | Describe method of data extraction from reports (e.g., piloted forms, independently, in duplicate) and any processes for obtaining and confirming data from investigators. | **2** |
| Data items | 11 | List and define all variables for which data were sought (e.g., PICOS, funding sources) and any assumptions and simplifications made. | **2** |
| Risk of bias in individual studies | 12 | Describe methods used for assessing risk of bias of individual studies (including specification of whether this was done at the study or outcome level), and how this information is to be used in any data synthesis. | **NA** |
| Summary measures | 13 | State the principal summary measures (e.g., risk ratio, difference in means). | **3** |
| Synthesis of results | 14 | Describe the methods of handling data and combining results of studies, if done, including measures of consistency (e.g., I2) for each meta-analysis. | **3** |
| Risk of bias across studies | 15 | Specify any assessment of risk of bias that may affect the cumulative evidence (e.g., publication bias, selective reporting within studies). | **3** |
| Additional analyses | 16 | Describe methods of additional analyses (e.g., sensitivity or subgroup analyses, meta-regression), if done, indicating which were pre-specified. | **3** |
| **RESULTS** | | |  |
| Study selection | 17 | Give numbers of studies screened, assessed for eligibility, and included in the review, with reasons for exclusions at each stage, ideally with a flow diagram. | **4** |
| Study characteristics | 18 | For each study, present characteristics for which data were extracted (e.g., study size, PICOS, follow-up period) and provide the citations. | **5-6** |
| Risk of bias within studies | 19 | Present data on risk of bias of each study and, if available, any outcome level assessment (see item 12). | **NA** |
| Results of individual studies | 20 | For all outcomes considered (benefits or harms), present, for each study: (a) simple summary data for each intervention group (b) effect estimates and confidence intervals, ideally with a forest plot. | **6-8** |
| Synthesis of results | 21 | Present results of each meta-analysis done, including confidence intervals and measures of consistency. | **6-8** |
| Risk of bias across studies | 22 | Present results of any assessment of risk of bias across studies (see Item 15). | **6-8** |
| Additional analysis | 23 | Give results of additional analyses, if done (e.g., sensitivity or subgroup analyses, meta-regression [see Item 16]). | **6-8** |
| **DISCUSSION** | | |  |
| Summary of evidence | 24 | Summarize the main findings including the strength of evidence for each main outcome; consider their relevance to key groups (e.g., healthcare providers, users, and policy makers). | **NA** |
| Limitations | 25 | Discuss limitations at study and outcome level (e.g., risk of bias), and at review-level (e.g., incomplete retrieval of identified research, reporting bias). | **NA** |
| Conclusions | 26 | Provide a general interpretation of the results in the context of other evidence, and implications for future research. | **NA** |
| **FUNDING** | | |  |
| Funding | 27 | Describe sources of funding for the systematic review and other support (e.g., supply of data); role of funders for the systematic review. | **NA** |

Table 3 Summary estimates of the proportion (%) of adverse pregnancy outcomes among women with untreated syphilis

| Adverse pregnancy outcomes | No. of studies | No. events | Total No. | Proportion, % (95% CI) | Heterogeneity | | Publication bias test | |
| --- | --- | --- | --- | --- | --- | --- | --- | --- |
|  |  |  |  |  | I^2^ (%) | p | t | p |
| Spontaneous abortion | 23 | 142 | 1,035 | 13.2 (10.2-16.4) | 47.5 | 0.006 | 1.464 | 0.158 |
| Stillbirth | 56 | 360 | 1,551 | 21.4 (17.7-25.3) | 66.5 | < 0.001 | -0.586 | 0.561 |
| Preterm birth/low birth weight | 34 | 260 | 1,388 | 17.6 (14.7-20.8) | 49.1 | 0.001 | -0.240 | 0.812 |
| Neonatal death | 19 | 89 | 948 | 8.2 (4.4-12.8) | 76.2 | 0.051 | 0.924 | 0.368 |
| Congenital syphilis | 39 | 504 | 1,447 | 34.0 (31.0-36.0) | 85.8 | < 0.001 | 0.365 | 0.717 |

Table 4 Summary estimates of the proportion (%) of adverse pregnancy outcomes among women with treated syphilis

| Adverse pregnancy outcomes | No. of studies | No. events | Total No. | Proportion, % (95% CI) | Heterogeneity | | Publication bias test | |
| --- | --- | --- | --- | --- | --- | --- | --- | --- |
|  |  |  |  |  | I^2^ (%) | p | t | p |
| Spontaneous abortion | 27 | 123 | 2,488 | 4.2 (2.9-5.8) | 58.4 | < 0.001 | 0.370 | 0.715 |
| Stillbirth | 45 | 235 | 5,831 | 3.1 (2.1-4.2) | 72.1 | < 0.001 | 0.286 | 0.777 |
| Preterm birth/low birth weight | 44 | 362 | 4,165 | 7.5 (5.7-9.5) | 79.5 | < 0.001 | 0.270 | 0.789 |
| Neonatal death | 19 | 21 | 3,713 | 0.4 (0.1-0.7) | 37.5 | 0.051 | -0.631 | 0.537 |
| Congenital syphilis | 53 | 577 | 6,286 | 8.8 (6.7-11.2) | 88.8 | < 0.001 | 1.932 | 0.059 |

Table 5 Summary estimates of the proportion (%) of adverse pregnancy outcomes among women without syphilis

| Adverse pregnancy outcomes | No. of studies | No. events | Total No. | Proportion, % (95% CI) | Heterogeneity | | Publication bias test | |
| --- | --- | --- | --- | --- | --- | --- | --- | --- |
|  |  |  |  |  | I^2^ (%) | p | t | p |
| Spontaneous abortion | 7 | 6 | 571 | 0.6 (0-1.6) | 0 | 0.446 | -0.659 | 0.539 |
| Stillbirth | 18 | 827 | 45,798 | 1.4 (0.7-2.2) | 92.4 | < 0.001 | 0.639 | 0.532 |
| Preterm birth/low birth weight | 16 | 2,483 | 45,271 | 4.7 (3.1-6.6) | 96.9 | < 0.001 | 0.505 | 0.623 |
| Neonatal death | 4 | 3 | 552 | 0.2 (0-0.9) | 0 | 0.812 | -1.015 | 0.417 |

**References**

1. Hao L. Effect of syphilis treatment during pregnancy on pregnancy outcomes and neonatal prognosis. Maternal and Child Health Care of China. 2016; 31: 3756-3758. doi: 10.7620/zgfybj.j.issn.1001 -4411.2016.18.39.

2. Meng F. Clinical research of poor pregnancy outcome of 204 cases with syphilis in pregnancy in eastern Henan. China Practical Medical. 2016; 11: 14-15. doi: 10.14163/j.cnki.11-5547/r.2016.21.007.

3. Wang L, Lin J, Wang X, Ji Z. Effect of drug therapy on the pregnancy outcomes of pregnant patients with syphilis. Chinese Journal of Human Sexuality. 2016; 25: 111-114. doi: 10.3969/j.issn.1672-1993.2016.08.037.

4. Yu J, Qi F, Li X. Treatment and maternal outcomes of 120 pregnant patients with syphilis. Chinese Journal of Human Sexuality. 2016; 25: 57-59. doi: 10.3969/j.issn.1672-1993.2016.09.018.

5. Zhang H, Fu H, Chen Q, Xiao B. Effect of anti-syphilis treatment and syphilis TRUST titer on the pregnant outcome of pregnant women with syphilis. Chinese Journal of Human Sexuality. 2016; 25: 122-124. doi: 10.3969/j.issn.1672-1993.2016.08.040.

6. Zhu J, Yu H. Analysis on impact of clinical intervention for syphilis in pregnant on pregnancy outcome. Chinese Journal of Experimental and Clinical Infectious Diseases (Electronic Version). 2016; 10: 363-366. doi: 10.3877/cma.j.issn.1674-1358.2016.03.024.

7. Cao D, Wang C. Clinical analysis on 72 offsprings of pregnant women with syphilis. Journal of Shanxi Medical College for Continuing Education. 2015; 25: 35-37.

8. Zeng X, Zhang X, Deng Y, Ye Y, Huang L, Wang W, et al. Clinical observation of the relationship between pregnant women with subclinical syphilis therapy and prognosis of perinatal infants. Modern Hospital. 2015; 15: 66-68, 70. doi: 10.3969/j.issn.1671-332X.2015.12.021.

9. Chen D, Li Z, Lin J, Zhang S, Wang F. Influence of standardized treatment to serofast pregnant women on gestation and infant serum. Chinese Journal of Nosocomiology. 2015; 25: 4525-4527. doi: 10.11816/cn.ni.2015-151033.

10. Chen F, Liao C, Huang X. Regression Analysis on the Effect of Early Intervention and Late Intervention of Gestational Syphilis in Pregnancy. Chinese Journal of Reproductive Health. 2015; 26: 166-168. doi: 10.3969/j.issn.1671-878X.2015.02.021.

11. Ding J. Clinical analysis of 86 cases of pregnancy complicated with syphilis. Maternal and Child Health Care of China. 2015; 30: 6207-6209. doi: 10.7620/zgfybj.j.issn.1001-4411.2015.35.17.

12. Huang M, Gu H. Impact of latent syphilis with late pregnancy on the maternal and infant and prevention countermeasures. Chinese Journal of Human Sexuality. 2015; 24: 90-93. doi: 10.3969/j.issn.1672-1993.2015.06.033.

13. Lian J, Huang R, Liu Z, Lai Y, Wu L. Clinical significance of early diagnosis and treatment of maternal syphilis. Maternal and Child Health Care of China. 2015; 30: 1048-1050. doi: 10.7620/zgfybj.j.issn.1001-4411.2015.07.24.

14. Ma Y, Chen W, Xia W. Impact of pregnancy on expression of syphilis antibody titer and research on treatment timing. Chinese Journal of General Practice. 2015; 13: 771-773. doi: 10.16766/j.cnki.issn.1674-4152.2015.05.025.

15. Wang C, Wang G, Han G, Jiang H, Ding W. Clinical analysis of pregnancy outcomes in 192 cases with syphilis. Jiangsu Medical Journal. 2015; 41: 1153-1155. doi: 10.19460/j.cnki.0253-3685.2015.10.013.

16. Xu Y, Shen X, Zhang C, Li X. Effect of intervention time on pregnancy outcomes among pregnant women with syphilis. Chinese Journal of AIDS and STD. 2015; 21: 418-420, 424. doi: 10.13419/j.cnki.aids.2015.05.18.

17. Yang H, Zhang Y. Clinical analysis of syphilis infection in pregnant women. China Tropical Medicine. 2015; 15: 210-212. doi:

18. Yuan Q. Effect of maternal syphilis on pregnancy outcomes. Practical Clinical Journal of Integrated Traditional Chinese and Western Medicine. 2015; 15: 52-53. doi: 10.13638/j.issn.1671-4040.2015.02.033.

19. Qin J, Feng T, Yang T, Hong F, Lan L, Zhang C, et al. Risk factors for congenital syphilis and adverse pregnancy outcomes in offspring of women with syphilis in Shenzhen, China: A prospective nested case-control study. Sex. Transm. Dis. 2014; 41: 13-23. doi: 10.1097/olq.0000000000000062.

20. Ding X, Huang Y, Yang L, Gu H. Adverse pregnancy outcomes of 265 pregnant women with latent syphilis. China Tropical Medicine. 2014; 14: 451-454. doi: 10.13604/j.cnki.46-1064/r.2014.04.017.

21. Ju L, Bai S, Zhang R, Ji Y. Prognosis analysis of 147 pregnant women with syphilis infection who had recived syphilis treatment in different periods during pregnancy. Yiayao Qianyan. 2014; 2014: 28-29. doi: 10.3969/j.issn.2095-1752.2014.20.024.

22. Lin K. Influence of Anti-syphilis treatment on pregnancy outcome and neonatal prognosis. West China Medical Journal. 2014; 29: 1267-1270.

23. Sun C, Zhang S. Analysis on the pregnancy outcome of 126 pregnant women with syphilis. International Journal of Epidemiology and Infectious Disease. 2014; 41: 41-43. doi: 10.3760/cma.j.issn.1673-4149.2014.01.010.

24. Sun Y, Su H, Ling J, Zhu Y, Tan J. Epidemiological characteristic and pregnancy outcomes of 105 cases of pregnancy complicated with syphilis. Chinese Journal of Woman and Child Health Research. 2014; 25: 758-760. doi: 10.3969/j.issn.1673-5293.2014.05.016.

25. Wang D, Chen J, Tang S. Analysis on impact of clinical interventions for syphilis during pregnancy on MTCT. Chinese Journal of Nosocomiology. 2014; 24: 4105-4107. doi: 10.11816/cn.ni.2014-133128.

26. Wang L, Feng L, Huang M, Gu H. Pregnancy outcome of patients with syphilis and its influence factors. Chinese Journal of Human Sexuality. 2014; 23: 47-49. doi: 10.3969/j.issn.1672-1993.2014.10.015.

27. Zhang C, Xu D, Liang P, Ding C. Study on anti-syphilis therapy for pregnant syphilis. Chinese Journal of Nosocomiology. 2014; 24: 4463-4465. doi: 10.11816/cn.ni.2014-132150.

28. Lin S, Wu X, Ye Z. Impact of syphilis infection during early pregnancy on pregnancy outcome. Chinese Journal of Nosocomiology. 2013; 23: 5225-5227.

29. Shi J, Ddiao Y, Li X. Study on the Pregnancy Outcome and Prognosis of Different Curative Opportunities on Pregnant Syphilis. The Chinese Journal of Dermatovenereology. 2013; 27: 274-276.

30. Wang F, Hong L. Study on pregnancy outcome and follow-up of pregnancy with sero-fixation of syphilis by TRUST and TPPA method. Maternal and Child Health Care of China. 2013; 28: 2044-2046. doi: 10.7620/zgfybj.j.issn.1001-4411.2013.28.10.

31. Wei H, Zhong Y. Treatment for pregnant syphilis and its effect on perinatal outcome. Journal of Hainan Medical University. 2013; 19: 381-383, 386. doi: 10.13210/j.cnki.jhmu.2013.03.012.

32. Wei H, Chen Z, Zeng Y, Zeng L, Jin Y. Effect of anti-syphilis treatment on pregnancy outcome. Chinese Journal of Experimental and Clinical Infectious Diseases (Electronic Version). 2013; 7: 83-85. doi: 10.3877/cma.j.issn.1674-1358.2013.04.025.

33. Xu X, Zhang G, Jiao T. An analysis of adverse pregnancy outcome and follow-up among pregnant women with syphilis treated with penicillin. Chinese Journal of AIDS and STD. 2013; 19: 438-440. doi: 10.13419/j.cnki.aids.2013.06.027.

34. Zhang X, Chen F. The relationship between syphilis treatment and perinatal outcomes. Journal of Preventive Medicine. 2013; 25: 72-75. doi: 10.19485/j.cnki.issn1007-0931.2013.11.026.

35. Zhou D, Xie H, Zhang Y, Hu Y. The effect of syphilis treatment in different pregnancy duration on pregnancy outcome. Journal of Diagnosis and Therapy on Dermato-venereology. 2013; 20: 121-123. doi: 10.3969/j.issn.1674-8468.2013.02.016.

36. Zhou M, Chen Z, Zeng Y, He K, Zhu Z, Huang H. Effect of anti-syphilis treatment on perinatal outcomes and neonatal prognosis in pregnant women complicated with syphilis. Chinese Journal of Clinical Infectious Diseases. 2013; 6: 226-229. doi: 10.3760/cma.j.issn.1674-2397.2013.04.009.

37. Liu Y. Effect of benzathine penicillin for pregnant women with syphilis on pregnancy outcomes. International Medicine and Health Guidance News. 2012; 18: 3144-3146. doi: 10.3760/cma.j.issn.1007-1245.2012.21.022.

38. Wang X, Li C, Liu X, Liu J. Effect of blocking of maternal-fetal transmission of syphilis on pregnancy and nursing. Chinese Journal of Modern Nursing. 2012; 18: 1277-1279. doi: 10.3760/cma.j.issn.1674-2907.2012.11.013.

39. Wang F, Lu J, Liang S, Lu D. Clinical analysis of 70 pregnant women with syphilis infection. Maternal and Child Health Care of China. 2012; 27: 4881-4883.

40. Xu Z, Qiu L, Li P, Zhu H, Liang Y, Shuai J, et al. Retrospective Study of 772 Pregnant Women on the Effectiveness of Mother-to-child Transmission-blocking of Syphilis. The Chinese Journal of Dermatovenereology. 2012; 26: 720-722.

41. Yang L, Hu M, Chen L. Clinical analysis of the screening and therapy for syphilis during the pregnancy. China Modern Doctor. 2012; 50: 150-151, 153. doi: 10.3969/j.issn.1673-9701.2012.17.067.

42. Zhang R, Kan N. Pregnancy outcome of women with syphilis. Anhui Medical and Pharmaceutical Journal. 2012; 16: 192-193. doi: 10.3969/j.issn.1009-6469.2012.02.021.

43. Li X, Liu H, Huang Y, Lin Y, Xu W. Survey and analysis of the blocking effectors between maternal and infant in pregnant women with syphilis. Experimental and Laboratory Medicine. 2011; 29: 345-346, 386. doi: 10.3969/j.issn.1674-1129.2011.04.004.

44. Li Z, Tian L, Luo Z, Zhou G, Yuan J, Yang Z. Correlation of pregnant syphilis with congenital syphilis. China Tropical Medicine. 2011; 11: 1383-1385. doi: 10.13604/j.cnki.46-1064/r.2011.11.035.

45. Wang M, Lyu J. Clinical analysis of 129 pregnant women with syphilis infection. Journal of Chinese Physician. 2011: 122-123. doi: 10.3760/cma.j.issn.1008-1372.2011.z1.060.

46. Zhang F. 156 cases of pregnancy outcomes of women infected with syphilis. Anhui Medical and Pharmaceutical Journal. 2011; 15: 1557-1558. doi: 10.3969/j.issn.1009-6469.2011.12.040.

47. Liu JB, Hong FC, Pan P, Zhou H, Yang F, Cai YM, et al. A risk model for congenital syphilis in infants born to mothers with syphilis treated in gestation: a prospective cohort study. Sex. Transm. Infect. 2010; 86: 292-296.

48. Zhu L, Qin M, Du L, Xie RH, Wong T, Wen SW. Maternal and congenital syphilis in Shanghai, China, 2002 to 2006. Int. J. Infect. Dis. 2010; 14: e45-e48. doi: 10.1016/j.ijid.2009.09.009.

49. Chen J. Relationship between treatment of syphilis during pregnancy and adverse pregnancy outcomes. Practical Preventive Medicine. 2010; 17: 1815-1816. doi: 10.3969/j.issn.1006-3110.2010.09.044.

50. Chen L. Analysis of Perinatal Results of 165 Cases with Syphilis during Pregnancy. Journal of Preventive Medicine. 2010; 22: 72-73. doi: 10.3969/j.issn.1007-0931.2010.06.036.

51. Jiao T, Xu X, Han C, Liu L, Zhou Y, Zhang G. Perinatal Outcomes of Pregnant Women with Syphllis Treated by Procaine Penicillin at Different Gestations. The Chinese Journal of Dermatovenereology. 2010; 24: 934-936.

52. Tang H, Hua Y, Wu J. Influence of Pregnancy Combined with Syphilis to Maternal-child. Chinese Journal of Misdiagnostics. 2010; 10: 27.

53. Zha D. Analysis of pregnany outcome among 45 pregnant women with syphilis infection. Chinese Journal of Family Planning. 2009: 299-300. doi: 10.3969/j.issn.1004-8189.2009.05.014.

54. Chen L. Clinical analysis of 75 pregnant women with syphilis infection. Medical Information. 2009; 22: 1105-1107. doi: 10.3969/j.issn.1006-1959-C.2009.12.013.

55. Huang Z, Zhou J, Luo R, Chen Y, Deng B, Liang R, et al. Effect of syphilis treatment before and after 28 gestational weeks on prognosis of newborns. China journal of Leprosy and Skin Diseases. 2009; 25: 355-357. doi: 10.3969/j.issn.1009-1157.2009.05.017.

56. Sun J, Yang H. Clinical analysis of 103 pregnant women with syphilis infection. Chinese Journal of Birth Health and Heredity. 2009; 17: 74-76. doi: 10.13404/j.cnki.cjbhh.2009.02.068.

57. Li C, Zhou K, Xing L. Effect of syphilis in pregnancy on pregnancy outcome. Modern Journal of Integrated Traditional Chinese and Western Medicine. 2008; 17: 1190-1191. doi: 10.3969/j.issn.1008-8849.2008.08.038.

58. Shao C. Clinical analysis of 202 cases of syphilis in pregnancy. Modern Journal of Integrated Traditional Chinese and Western Medicine. 2008; 17: 3598-3599. doi: 10.3969/j.issn.1008-8849.2008.23.018.

59. Sun L, Chen Y, Zhang P. Penicillin treatment of syphilis in pregnancy and its pregnancy outcome. Chinese Journal of Birth Health and Heredity. 2008; 16: 80-81. doi: 10.3969/j.issn.1006-9534.2008.06.040.

60. Tan X, Liu H, Xiong H, Ning J, Li B, Peng G. Study on the timing of blocking maternal-fetal transmission of syphilis during pregnancy. Chinese Journal of Reproductive Health. 2008; 19: 199-202. doi: 10.3969/j.issn.1671-878X.2008.04.003.

61. Xiong H. Clinical analysis of 71 cases of pregnant syphilis. Guide of China Medicine. 2008; 6: 38-41. doi: 10.3969/j.issn.1671-8194.2008.11.020.

62. Lai Y, Yang F, Hong F, Wang L, Yan D, Lin L. Syphilis in pregnancy women. Chinese Journal of Primary Medicine and Pharmacy. 2007; 14: 49-50. doi: 10.3760/cma.j.issn.1008-6706.2007.01.025.

63. Lyu X, Fan J, Meng Z. A retrospective analysis of pregnancy associated with syphilis. Chinese Journal of Woman and Child Health Research. 2007; 18: 457-459. doi: 10.3969/j.issn.1673-5293.2007.05.040.

64. Wang X, Liu C, Xiang X. Analysis of relationship between treatment of gestational patients complicated with syphilis and perinatal prognosis. China Tropical Medicine. 2007; 7: 951. doi: 10.3969/j.issn.1009-9727.2007.06.047.

65. Yuan Y, Yuan H, Huang Q. Clinical analysis of 128 cases of pregnancy syphilis. Guangxi Medical Journal. 2007; 29: 1446-1447. doi: 10.3969/j.issn.0253-4304.2007.09.079.

66. Zhang L, Chen Q, Chen L, Wang X, Zhang L, Xiu X, et al. Study on interventional methods and the pattern of maternal-fetal transmission of syphilis during pregnancy. Chinese Journal of Obstetrics and Gynecology. 2007; 42: 438-442. doi: 10.3760/j.issn:0529-567x.2007.07.003.

67. Zhang Y. Clinical analysis of 137 cases of pregnant syphilis. Chinese Journal of Rural Medicine and Pharmacy. 2007; 14: 37-38. doi: 10.3969/j.issn.1006-5180.2007.06.030.

68. Chen L. Clinical analysis of pregnant women with syphilis. Journal of Tropical Medicine. 2006; 6: 406-408. doi: 10.3969/j.issn.1672-3619.2006.04.016.

69. Deng Q, Wen J, He Q, Deng Q. Effects of anti-syphilis treatment during pregnancy on fetus. Chinese Journal of Primary Medicine and Pharmacy. 2006; 13: 846-847. doi: 10.3760/cma.j.issn.1008-6706.2006.05.086.

70. Hu B, Zeng X, Deng X, Xie J, Tang Y. Relationship between treatment of pregnancy complicated syphilis and the prognosis of infant. The Journal of Practical Medicine. 2006; 22: 1861-1863. doi: 10.3969/j.issn.1006-5725.2006.16.010.

71. Huang X. Clinical diagnosis and treatment of 87 cases of maternal syphilis. Guangdong Medical Journal. 2006; 27: 739-740. doi: 10.3969/j.issn.1001-9448.2006.05.065.

72. Xuan Q, Zhang X, Jiang M, Xiao X, Zhou X. Perinatal outcomes of 286 pregnant women with syphilis treated by benzylpenicillin at different gestations. Chinese Journal of Perinatal Medicine. 2006; 9: 400-403. doi: 10.3760/cma.j.issn.1007-9408.2006.06.012.

73. Zhu H, Gu J, Su X, Lai W. Effect of Treatment of Syphilis During Pregnancy and Pre-pregnancy on Pregnant Outcome. Journal of Sun Yat-sen University (Medical Sciences). 2006; 27: 200-202. doi: 10.3321/j.issn:1672-3554.2006.02.020.

74. Fang H, Luo R. The effects of syphilis during pregnancy on pregnant outcomes. Hainan Medical Journal. 2005; 16: 36-37. doi: 10.3969/j.issn.1003-6350.2005.10.020.

75. Li Q, Huang S, Wang J. Affects on gravidas and neonates in cases of late pregnancy complicating with latent syphilis. Maternal and Child Health Care of China. 2005; 20: 1477-1478. doi: 10.3969/j.issn.1001-4411.2005.12.030.

76. Zhang X, He P. Clinical analysis and pregnany outcome of syphilis during pregnancy. Guangdong Medical Journal. 2005; 26: 806-807. doi: 10.3969/j.issn.1001-9448.2005.06.039.

77. Kuang Y. The significance of syphilis serial test of pregnant women. Modern Hospital. 2004; 4: 49-50. doi: 10.3969/j.issn.1671-332X.2004.04.026.

78. Wang C, Zheng J. Relationship between maternal syphilis and pregnancy outcome. Practical Clinical Journal of Integrated Traditional Chinese and Western Medicine. 2004; 4: 61-62. doi: 10.3969/j.issn.1671-4040.2004.05.063.

79. Zhang X, Zhang R, Lin S, Chen S, Zheng L. Clinical analysis of 192 pregnant women infected by syphilis. Chinese Journal of Obstetrics and Gynecology. 2004; 39: 38-42. doi: 10.3760/j.issn:0529-567X.2004.10.010.

80. Lin X, Wu J, Wen J. Clinical analysis of 41 cases of syphilis during pregnancy. Chinese Journal of Reproductive Health. 2002; 13: 3-5. doi: 10.3969/j.issn.1671-878X.2002.01.002.

81. Liu B, Zheng W. Clinical analysis of pregnancy outcome in pregnant women infected by syphilis during pregnancy. Chinese Journal of Primary Medicine and Pharmacy. 2002; 9: 20-21. doi: 10.3760/cma.j.issn.1008-6706.2002.11.009.

82. Chen S, Zheng B, Yang B. Effect of syphilis in pregnancy on fetus. Chinese Journal of Practical Gynecology and Obstetrics. 2001; 17: 49-50. doi: 10.3969/j.issn.1005-2216.2001.03.024.

83. Lyu J, Yang X, Chen C, Huang C, Kong X, Zeng Y, et al. Sexual Transmitted Disease in Pregnancy Syphilis in pregnancy women. Chinese Journal of Obstetrics and Gynecology. 2001; 36: 456-459. doi: 10.3760/j.issn:0529-567X.2001.08.002.
